# Complex immune responses and molecular reactions to pathogens and disease in a desert reptile (*Gopherus agassizii*)

**DOI:** 10.1002/ece3.4897

**Published:** 2019-02-18

**Authors:** K. Kristina Drake, Christina M. Aiello, Lizabeth Bowen, Rebecca L. Lewison, Todd C. Esque, Kenneth E. Nussear, Shannon C. Waters, Peter J. Hudson

**Affiliations:** ^1^ Western Ecological Research Center U.S. Geological Survey Henderson Nevada; ^2^ Department of Biology San Diego State University San Diego California; ^3^ Graduate Group in Ecology University of California‐Davis Davis California; ^4^ Department of Biology Pennsylvania State University University Park Pennsylvania; ^5^ Western Ecological Research Center U.S. Geological Survey Davis California; ^6^ Department of Geography University of Nevada‐Reno Reno Nevada

**Keywords:** desert tortoise, immunity, mRNA, *Mycoplasma agassizii*, transcription

## Abstract

Immune function plays an important role in an animal's defense against infectious disease. In reptiles, immune responses may be complex and counterintuitive, and diagnostic tools used to identify infection, such as induced antibody responses are limited. Recent studies using gene transcription profiling in tortoises have proven useful in identifying immune responses to various intrinsic and extrinsic stressors. As part of a larger experiment with Mojave desert tortoises (*Gopherus agassizii*), we facilitated the transmission of the pathogenic bacteria, *Mycoplasma agassizii *(Myag), to naïve adults and measured innate and induced immune reactions over time. Specifically, we evaluated clinical condition, presence of Myag in the nasal/oral cavity, induced antibody responses specific to Myag, and measured molecular reactions (gene transcript profiles) in 15 captive tortoises classified as naïve, exposed, or infected and 14 wild tortoises for comparison. Myag was confirmed inside the nasal/oral cavity in exposed tortoises within 30–60 days of introduction to infected animals, yet we did not detect Myag specific induced antibody responses in these individuals until 420–595 days post exposure. Surprisingly, we found no overall differences in the gene transcript profiles between our experimental treatment groups throughout this study. This work highlights the complexities in assessing immune function and diagnosing pathogen related infections in tortoises and other reptiles.

## 1 INTRODUCTION

The immune response is central in the defenses against infectious disease, but accurately measuring immune function and targeted responses in nonmodel wildlife can be challenging (Bowden, Thompson, Morgan, Gratacap, & Nikoskelainen, 2007; Matson, Cohen, Klasing, Ricklefs, & Scheuerlein, 2006; Zimmerman, Vogel, & Bowden, 2010). The vertebrate immune system is a complex network of organs, tissues, circulating cells, and molecules that include both innate and induced (adapted) mechanisms (Ellis, 2012). Extensive biomedical studies on humans and related species have greatly improved our understanding of how this system works (Brodin & Davis, 2017; Demas & Nelson, 2012) and helped guide the development of diagnostic assays and biomarkers used to measure immune responses to pathogens and diseases. Conversely, immune function in nonmammalian vertebrates has not been well studied (Bowden et al., 2007; Uller, Isaksson, & Olsson, 2006; Zimmerman, Paitz, Vogel, & Bowden, 2010), and their immune responses are likely influenced by many factors including metabolic capacity (Chen, Cuijuan, & Pu, 2007; Hsu, 1998), endocrine fluctuations (Martin, Weil, & Nelson, 2008), season (Bowden et al., 2007; Munoz & De la Fuente, 2001; Sandmeier, Horn, & Tracy, 2016), temperature (Goessling et al., 2017; Zapata, Varas, & Torroba, 1992), and other environmental conditions (Martin et al., 2008; Origgi, 2007).

Reptiles, like most ectotherms, invest in, and rely on broad innate responses such as nonspecific leukocytes, lysozymes, antimicrobial peptides, the complement pathway, and behaviorally induced fever as their primary lines of defense against pathogens (Rios & Zimmerman, 2015; Zimmerman, Paitz, et al., 2010). These responses are germline encoded and are relatively rapid and nonspecific. Adaptive immune reactions, such as T and B cells are produced in reptiles; however, their cell‐mediated and humoral responses may be slower and much less robust than avian or mammalian responses (Aiello et al., 2016; Maloney, 2011; Origgi, 2007) or may fail to develop (Sandmeier, Tracy, DuPre, & Hunter, 2012). Reptiles lack lymph nodes and therefore do not form germinal centers, the site typically associated with somatic hypermutation and affinity maturation of B cells in mammals (Hsu, 1998; Janeway, Travers, Walport, & Schlomick, 2005; Snoeijs, Eens, Steen, & Pinxten, 2007). Antibody production by selected plasma cells also requires high rates of metabolism (Alberts et al., 2002) and is energetically expensive (Sandmeier & Tracy, 2014). Consequently, ectothermic reptiles with reduced metabolic activity and limited resources likely rely more on innate immunity, selected cells such as phagocytic B cells, or natural antibody responses for their protection from pathogens and infectious disease (Hunter, Dupre, Sharp, Sandmeier, & Tracy, 2008; Rios & Zimmerman, 2015; Zimmerman, Paitz, et al., 2010; Zimmerman, Vogel, Edwards, & Bowden, 2009). Immune responses in reptiles may also vary depending on the type of pathogen (bacteria, virus, multicellular parasite, etc.) and other modifiers (dose/intensity, virulence, route, prior exposure; Power, Wei, & Bretscher, 1998, Goldsby, Kindt, Kuby, & Osbourne, 2003; Adamo, 2004). Moreover, small reductions in some elements of immune function can lead to significant increases in disease susceptibility, whereas larger reductions in other elements seem to have little effect (Keil, Luebke, & Preuett, 2001).

Wildlife studies on health and epidemiological patterns often focus on adaptive responses (e.g., indicators of targeted antibody response such as seroconversion, seroprevalence)—as a means to understand infection patterns and disease impacts on wildlife (Rodgers, Toline, & Rice, 2018). However, given the limited understanding of immunity in reptiles (Zimmerman, Vogel, et al., 2010) and the additional time and resources needed to elicit an induced response (Sandmeier, Weitzman et al., 2017), a broader approach may be more appropriate to assess their responses to infection or other stressors. Recent studies using gene transcription profiling have advanced the evaluation of immune function in reptiles (Bowen et al., 2015; Drake et al., 2017, 2016; Krivoruchko & Storey, 2013, 2015) and other ectotherms (Connon et al., 2012). Gene transcription works by targeting specific genes that respond to intrinsic (e.g. pathogens) or extrinsic (e.g. environmental contaminants) stressors and measures their responses by quantifying the amount of messenger RNA (mRNA) that is transcribed (Bartosiewicz, Penn, & Buckpitt, 2001; Bowen et al., 2012; Burczynski et al., 2000; Miles et al., 2012; Sitt et al., 2008). This approach incorporates multiple genes that can be used to detect early, observable signs of physiological changes at the cellular level (Acevedo‐Whitehouse & Duffus, 2009).

To better understand immune responses to pathogenic infections in chelonians, we studied the Mojave desert tortoise (*Gopherus agassizii*), a longlived herbivorous reptile that occurs throughout the Mojave Desert, USA. Mojave desert tortoises are currently listed as Threatened under the Endangered Species Act and an infectious disease (upper respiratory tract disease—URTD) has been named as a factor in their decline (USFWS, 1994, 2011). URTD is common among chelonians in North America and Europe, and is characterized by mild to severe rhinitis, nasal and ocular discharge, conjunctivitis, periocular edema, lethargy, and occasionally death in conjunction with other complicating factors (Brown et al., 1994; Jacobson et al., 2014; Origgi & Jacobson, 2000; Sandmeier, Tracy, duPré, & Hunter, 2009). In most tortoises and some turtles, URTD is primarily caused by the bacteria, *Myocplasma agassizii* (Myag; Brown et al., 1994; Brown et al., 1999; Palmer, Blake, Wellehan, Childress, & Deem, 2016; Sandmeier, Weitzman et al., 2017) and to a lesser extent *M. testudineum* (Myte; see reviews in Sandmeier et al., 2009; Jacobson et al., 2014).

We quantified innate and induced immune responses using gene transcript profiles (Bowen et al., 2015) and measured induced antibody levels for *Mycoplasma* spp. with traditional assays (Brown et al., 2002; Wendland et al., 2007) in captive and wild Mojave desert tortoises over time. We investigated the presence and timing of immune responses after exposure to Myag and whether transcript profiles for genes involved in immune responses to pathogenic microbes (*SAA, ATF, CD9, MX1, MyD88;* Zhou, Guo, & Dai, 2008; Zhou, Wang, Feng, Guo, & Dai, 2011; Kibenge, Munir, & Kibenge, 2005; Tumpey et al., 2007; Li et al., 2011) and genes often correlated with malnutrition (*Lep;* Otero et al., 2005) and cellular stress (*SOD*; Walsh, Leggett, Carter, & Colle, 2010) would be higher in tortoises exposed to Myag and suspected of having bacteria‐related infection and disease (Bowen et al., 2015; Drake et al., 2017). Our research asks how molecular biomarkers can improve and expedite diagnosis of immune responses and diseases in tortoises, and advance reptile ecoimmunology.

## 2 METHODS

### 2.1 Study animals and experimental design

Captive adult male Mojave desert tortoises (*G. agassizii*; *n* = 15) were studied at the Desert Tortoise Conservation Center in Clark County, Nevada, USA. Each tortoise was housed in an outdoor enclosure (232.3 m^2 ^or 0.02 ha; *n* = 10) with native perennial vegetation, artificial and natural burrows, and irrigation systems that provided water every 4 days. Tortoises were fed a commercial diet (Zoo Med Natural Grassland Tortoise Food®, San Luis Obispo, California) biweekly during periods of activity (March–October). Annual grass and forb food plants that germinated from native seed‐bank were also available periodically in each enclosure throughout the experiment. Tortoises were housed individually and visually examined for health and disease presence by biologists and veterinarians with extensive desert tortoise medical and management experience for six months prior to our study.

Captive tortoises with chronic URTD were selected as “infected” tortoises (*n* = 5) and used to facilitate disease transmission to uninfected tortoises under natural conditions (Aiello et al., 2016). Infected tortoises were defined by the presence of clinical signs associated with URTD (periocular swelling, ocular and/or nasal discharge, respiratory distress), seropositive for induced antibodies to Myag using an enzyme‐linked immunosorbant assay, and presence of Myag in the oral cavity confirmed via qPCR (Aiello et al., 2016). Uninfected tortoises (*n* = 10) were classified as clinically normal, and negative for serological immune responses and presence of Myag and Myte in the oral cavity. Tortoises were evaluated and sampled monthly for six months prior to our experiment to confirm the clinical, immunological, and infection condition of each animal (Aiello, Esque, Nussear, Emblidge, & Hudson, 2018; Aiello et al., 2016). On 16 August 2013, one infected tortoise was randomly assigned and added to one of five enclosures housing two uninfected tortoises (hereafter referred to as “exposed” tortoises). The remaining five naïve uninfected tortoises were housed individually and isolated from other tortoises at the DTCC and will be referred to as “control” tortoises hereafter.

For comparison, a group of “reference” uninfected wild adult Mojave desert tortoises (*n* = 14; 8M:6F) were evaluated and sampled from an in situ population in Hidden Valley, Clark County, Nevada, USA (Drake et al., 2015). Reference tortoises were deemed clinically normal based on visual examination and free of *Mycoplasma* spp. infection for nine consecutive years (Drake et al., 2015). All animals were evaluated 180 days before and 222 days after the experiment. Due to the logistical constraints associated with closing of the DTCC (430 days post experiment), only exposed and reference tortoises were evaluated for an additional 500 days (722 post experiment). All handling and experiments using animals were conducted according to the Institutional Animal Care and Use Committee guidelines (U.S. Geological Survey WERC #2012‐03 and Pennsylvania State University IACUC #38532) and under the appropriate state (Nevada Division of Wildlife Permit #S33762) and federal (U.S. Fish and Wildlife Service TE‐030659) permits.

### 2.2 Animal condition

All captive tortoises were assessed monthly (2013) and then weekly (2014) to characterize their general health and body condition during periods of activity (March–October) between March 2013 and July 2014. Weekly assessments continued for exposed tortoises through October 2016. Health and body condition were evaluated seasonally (spring, summer, fall) each year for wild reference tortoises. Assessments included an examination of the animal's general posture, respiration, face (with specific attention to the eyes, periocular tissue, nares, mouth, tongue, and oral mucosa), skin, and shell for any clinical signs of disease, abnormalities, damage, or discoloration (USFWS, 2016). We looked for discharge from the cloaca, eyes, nares, and mouth and examined the skin for evidence of ulceration, erythema, swelling, or discharge (USFWS, 2016).

Numerical body condition scores (BCS) were used to assess the overall muscle condition and fat stores with respect to skeletal features of the head and limbs (USFWS, 2016). BCS scores were first categorized as “under”, “adequate”, or “over” condition, and then numerical values were assigned to provide a precise and repeatable measurement (i.e. Under: 1–3, Adequate: 4–6, Over: 7–9; USFWS, 2016).

### 2.3 Tissue collection

Immediately following the physical assessment, blood (~1 ml) was extracted via subcarapacial venipuncture (Hernandez‐Divers, Hernandez‐Divers, & Wyneken, 2002) using a 3.81‐cm, 23‐gage needle and 3 ml syringe coated in sodium heparin. Aliquots of whole blood were placed immediately into an RNeasy^®^ Animal Protect collection tube (0.5 ml blood; Qiagen, Valencia, CA) and BD Microtainer^®^ tubes with lithium heparin (remaining blood; Becton Dickinson and Company, Franklin Lakes, NJ). Samples were stored on ice in the field for no more than four hours. Plasma was separated from the remaining blood sample using centrifugation with a force of 1,318 × *g* and frozen at −80°C until analysis. Aliquots of plasma (0.05 ml) were shipped to the Mycoplasma Laboratory at the University of Florida (Gainesville, FL, USA) and screened for antibodies to Myag and Myte using an enzymelinked immunosorbant assay (ELISA measuring immunoglobulin M (IgM) and IgY light chains; Wendland et al., 2007). Results from ELISA were reported as negative (antibody titer <32), suspect (antibody titer ≥32 and <64), or positive (antibody titer ≥64).

Sloughed epithelial cells were collected using nasal and oral swabs (USFWS, 2016). Nasal swabs were collected using a small sterile polyester swab by rotating the swab tip approximately 2 mm inside and around the periphery each naris while slowly spinning the swab. Oral swabs were collected using two sterile polyester swabs side‐by‐side while slowing spinning the swab tips across the surfaces of the tongue and oral mucosa in one full rotation. Each swab tip was placed into a cryogenic vial and stored on ice while in the field. Additionally, nasal lavages were conducted on each animal by flushing 2.5 ml sterile 0.9% saline solution into each naris using a sterile 5 ml syringe and collecting the fluid exiting the opposite naris in a sterile conical vial. After swirling the collected fluid, we used a sterile pipette to transfer 1 ml of flush to a vial containing 200 μl of RNAlater RNA stabilizing reagent (Qiagen, Hilden, Germany), which was then stored on ice (Aiello et al., 2016). Nasal swabs, oral swabs, and lavage flushes were frozen to −80°C and then shipped on dry ice to the San Diego Zoo Amphibian Disease Lab (Escondido, CA, USA) to detect and estimate the abundance of Myag and Myte using quantitative polymerase chain reaction (qPCR) (Braun et al., 2014). Results for a qPCR test were reported as negative, positive, or equivocal (inconclusive). A positive result included three estimates of Myag or Myte abundance. We used the mean of these values in the analyses and results.

### 2.4 Gene transcription

RNA extractions and cDNA synthesis from blood were performed as described by Bowen et al. (2015) on each sample collected for gene transcription. PCR primers developed for *G. agassizii* were used to amplify 11 genes of interest and one ribosomal housekeeping gene within each sample (see Bowen et al., 2015 and Drake et al., 2016). Gene transcription cycle threshold values (*C_T_*) were measured for the housekeeping gene (18S) and the genes of interest: *AHR*‐Arylhydrocarbon Receptor, *ATF*, *CaM*‐Calmodulin, *CD9*, *CL*‐Cathepsin L, *HSP70*‐Heat Shock Protein 70, *Lep*‐Leptin, *Mx1*, *MyD88*‐Myeloid Differentiation Factor 88, *SAA*‐Serum Amyloid A, and *SOD*‐Superoxide dismutase from each sample in duplicate using quantitative PCR (Supporting Information Table S1). Amplifications were conducted on a StepOnePlus™ Real‐Time PCR System (Thermo Fisher Scientific, Hanover Park, IL). Gene transcription measures were normalized by subtracting the average 18S housekeeping ribosomal gene C_T_ value from the gene of interest *C_T_* for each tortoise.

We analyzed the qPCR gene transcript data using normalized *C_T_* values. These values are inversely proportional to the amount of subject mRNA in the sample such that the lower the normalized value, the more transcripts are present. A change of 2 in the normalized value is approximately equivalent to a fourfold change in the amount of the transcript. To examine the potential differences in immune function between the experimental treatment groups, we evaluated normalized gene transcript profiles for each gene of interest from each tortoise group (control, exposed, infected, reference), season, and sampling period throughout the study (Table 1). Sampling dates were: *Sample 1* in late July 2013 (22–45 days prior to experiment); *Sample 2* in early September 2013 (20 days post experiment); *Sample 3* in early November 2013 (77 days post experiment); and *Sample 4* in late March 2014 (222 days post experiment). Transcript profiles for exposed and reference tortoises were also analyzed during *Sample 5* in October 2014 (420 days post experiment).

**Table 1:** Data range and geometric mean normalized cycle threshold (*C_T_*) transcription values for 11 genes of interest for 15 adult captive male tortoises and 14 adult wild tortoises (8M:6F) in Clark County, Nevada, USA

Cycle Threshold (*C_T_*) Values											
Gene	Treatment	Preexperiment		Postexperiment							
		Sample 1		Sample 2		Sample 3		Sample 4		Sample 5	
		Range	Geo. mean	Range	Geo. mean	Range	Geo. mean	Range	Geo. mean	Range	Geo. mean
AHR	Control	8.44–14.86	12.51	2.82–15.73	10.74	11.92–14.80	13.75	10.05–13.71	12.17	—	—
	Exposed	12.46–16.15	13.65	12.76–16.56	14.43	13.04–16.60	14.02	14.09–16.27	14.81	13.72–14.63	14.00
	Infected	12.88–14.68	13.95	12.97–14.69	13.31	11.00–15.07	13.68	13.73–14.68	14.38	—	—
	Reference	11.24–16.64	14.25	—	—	12.55–15.83	14.29	12.34–16.02	14.52	12.90–16.24	14.48
ATF	Control	6.13–10.56	8.42	2.82–10.26	6.99	5.51–9.37	6.94	4.09–16.31	7.79	—	—
	Exposed	6.25–12.72	9.12	6.41–13.86	10.04	6.14–13.67	9.12	8.32–14.15	10.59	8.83–11.49	10.07
	Infected	7.14–10.10	9.05	8.19–10.18	9.58	6.12–11.14	9.31	11.07–12.04	11.42	—	—
	Reference	6.18–13.89	10.48	—	—	6.55–14.96	9.1	4.90–9.41	7.47	9.00–16.77	11.52
CaM	Control	8.75–9.90	9.36	4.30–10.34	8.24	7.01–10.91	8.51	6.88–13.26	8.99	—	—
	Exposed	7.93–11.00	8.78	8.38–11.88	9.66	8.63–11.79	9.84	8.79–11.90	10.12	8.52–10.23	9.20
	Infected	6.67–9.03	8.19	8.15–9.38	8.65	7.76–10.83	9.52	9.57–11.60	10.21	—	—
	Reference	7.54–11.97	9.53	—	—	6.57–11.27	8.92	7.49–11.06	9.57	8.77–11.61	9.93
CD9	Control	8.86–14.66	11.81	9.97–13.79	12.12	9.80–13.49	11.22	4.31–14.09	9.57	—	—
	Exposed	8.60–14.67	11.62	9.01–15.84	12.78	9.97–13.09	11.40	11.51–15.30	13.19	12.48–13.52	13.07
	Infected	10.09–13.01	11.78	10.82–12.75	11.66	9.41–12.55	11.25	11.81–13.78	13.23	—	—
	Reference	9.80–14.56	12.12	—	—	9.71–13.85	11.64	8.99–14.01	11.63	10.49–13.80	11.81
CL	Control	15.15–17.30	15.93	2.86–16.51	11.32	11.83–15.53	13.27	11.34–26.43	14.89	—	—
	Exposed	13.27–17.29	14.72	13.46–20.25	15.69	12.82–17.16	14.46	14.63–16.83	15.58	14.22–15.44	14.87
	Infected	12.19–15.56	14.41	13.29–15.26	14.44	10.78–15.41	13.98	15.55–16.70	15.96	—	
	Reference	13.19–18.97	15.85	—	—	10.05–21.82	15.34	12.50–16.29	14.77	14.00–22.88	16.50
HSP70	Control	9.98–13.07	11.47	3.00–13.69	9.63	10.87–12.71	12.16	10.48–12.61	11.58	—	—
	Exposed	11.41–12.93	11.93	10.98–13.29	12.28	11.94–13.39	12.59	11.90–13.90	12.84	12.39–12.97	12.61
	Infected	10.93–11.63	11.36	11.53–12.72	11.83	11.33–12.92	12.36	12.04–13.15	12.52	—	—
	Reference	9.36–13.85	12.33	—	—	10.94–14.64	12.69	11.47–13.75	12.57	11.98–13.75	12.71
Lep	Control	10.90–13.16	12.31	13.05–32.00	13.93	10.68–13.07	12.29	10.70–12.46	11.76	—	—
	Exposed	10.67–14.13	11.98	11.04–16.22	13.16	12.04–15.22	12.98	12.76–14.81	13.47	11.40–13.86	12.22
	Infected	10.73–14.08	12.65	10.83–13.31	11.79	10.07–14.45	12.75	12.83–13.65	13.39	—	—
	Reference	9.77–15.64	12.78	—	—	11.07–15.50	13.13	11.52–14.57	12.97	11.96–16.13	13.87
MyD88	Control	12.83–16.77	14.82	4.12–15.98	11.63	13.87–16.13	14.75	9.18–16.30	13.73	—	—
	Exposed	13.76–15.76	14.68	14.28–17.00	15.45	14.44–16.25	15.16	15.25–16.48	15.93	14.73–16.33	15.42
	Infected	13.56–15.31	14.71	13.81–15.17	14.47	12.89–15.95	14.73	14.99–16.07	15.58	—	—
	Reference	12.80–18.04	15.64	—	—	13.64–16.99	15.33	15.06–16.70	15.88	14.31–16.89	15.49
Mx1	Control	9.79–17.74	14.62	11.14–32.00	15.92	17.36–19.09	17.97	10.62–18.21	15.49	—	—
	Exposed	15.59–19.88	17.02	14.93–21.02	17.82	14.85–22.39	18.13	16.50–21.07	18.67	16.09–19.39	17.42
	Infected	14.78–20.08	18.11	15.11–17.00	15.17	14.48–20.28	17.59	16.13–18.93	17.43	—	—
	Reference	13.46–21.74	17.39	—	—	14.94–21.34	18.52	12.78–21.75	18.92	14.77–21.93	18.62
SAA	Control	6.62–15.94	12.07	6.62–17.45	13.69	14.73–17.04	15.74	7.96–15.56	11.89	—	—
	Exposed	13.76–16.55	15.05	12.88–20.39	15.93	13.59–18.30	15.01	14.54–18.37	16.11	14.38–16.45	15.36
	Infected	13.71–18.39	16.46	13.74–17.04	14.84	10.93–18.92	15.13	13.45–16.64	15.39	—	
	Reference	12.38–18.65	15.77	—	—	13.56–20.72	16.92	11.59–18,67	16.46	13.39–19.52	16.43
SOD	Control	6.47–12.10	9.52	8.04–11.42	10.32	7.64–11.35	8.61	2.91–12.06	7.46	—	—
	Exposed	7.94–11.28	9.41	8.70–13.26	10.75	8.08–12.00	9.42	9.86–12.6	10.98	9.51–11.25	10.38
	Infected	7.99–10.27	9.44	8.94–10.61	9.63	6.66–10.49	9.06	10.32–11.11	10.65	—	—
	Reference	7.48–14.70	10.63	—	—	6.49–11.35	8.97	7.10–10.76	9.11	7.87–11.74	9.55

Note

Tortoises include five control, five exposed, and four infected captive animals and 14 wild reference tortoises. Note the smaller the mean value, higher the level of transcript for 11 genes. Sampling occurred 22–45 days preexperiment in Jul 2013 (Sample 1), 20 days post in Sept 2013 (Sample 2), 77 days post in Nov 2013 (Sample 3), 222 days post in Mar 2014 (Sample 4), and 420 days post experiment in October 2014 (Sample 5).

—: Information is not available.

### 2.5 Statistical analyses

Counts of laboratory results for plasma ELISA, nasal and oral swabs, and nasal lavage tests were calculated for each individual for both Myag and Myte. Mean proportions of positive and/or suspect results were calculated for each treatment group during each week evaluated. Most gene transcript profiles were not normally distributed even after log transformations; therefore we used nonparametric tests for analyses. We evaluated the *C_T_* value for genes during each sampling event as well as the change (Δ*C_T_*) in transcript values between the first and final samples. The geometric means were calculated for the normalized cycle threshold (*C_T_*) transcription values for 11 genes of interest for tortoises in each treatment group. We used conventional mean responses per treatment group (control, exposed, infected, reference), and sampling period (1, 2, 3, or 4) with data assessed for statistical significance between classification ranks using nonparametric KruskalWallis rank sums tests (Kruskal & Wallis, 1952; R package stats v3.2.2). When a significant result warranted further inspection, we performed a posthoc Dunn's test with sequential Bonferonni corrections for multiple testing (Dunn, 1964; R package dunn.test v1.3.5; Dinno, 2015).

Gene transcript responses between the treatment groups and sampling period were compared using a nonparametric Multivariate Analysis of Variance (permutation MANOVA; R package vegan v2.3‐1). In addition, we evaluated potential influences of tortoise sex on gene transcript levels in our reference population using a permutation MANOVA. We also performed a nonmetric, multivariate, multidimensional scaling (NMDS; R package vegan v2.3‐1; Oksanen et al., 2011) ordination with the BrayCurtis similarity measure in conjunction with cluster analysis for statistical and graphical representation of individual tortoises clustered by similarity in transcription, and not by predefined groups such as experimental treatment. All statistical tests were conducted using R v3.3.1 (R Development Core Team, 2017) and significance was based on *p* values ≤0.05.

In an effort to fully explore the genetic patterns within our dataset, we also evaluated transcription profiles among treatment groups (control‐C, exposed‐E, infected‐I, reference‐R) by excluding reference (CEI) and infected (CE) tortoises as well as excluding control (EIR) and infected (ER) animals during each sampling period as described above. Reference individuals represent wild tortoises that were not provided supplemental food and water, possibly contributing to their transcription profiles throughout the experiment. However, reference tortoises have been thoroughly evaluated for health for 11 years and appear to represent a robust healthy population (Drake et al, 2015) making them a reasonable selection for inclusion. Control tortoises were initially selected for use in this experiment because they were clinically normal and without detectable levels of *Mycoplasma* spp. infection; however, aspects of their health were evaluated for only 6 months prior to our experiment. As such, we explored the idea of combining reference and control tortoises (CE) and excluding control tortoises from this experiment (groups EIR and ER).

## 3 RESULTS

### 3.1 Physical condition

**Figure 1:**
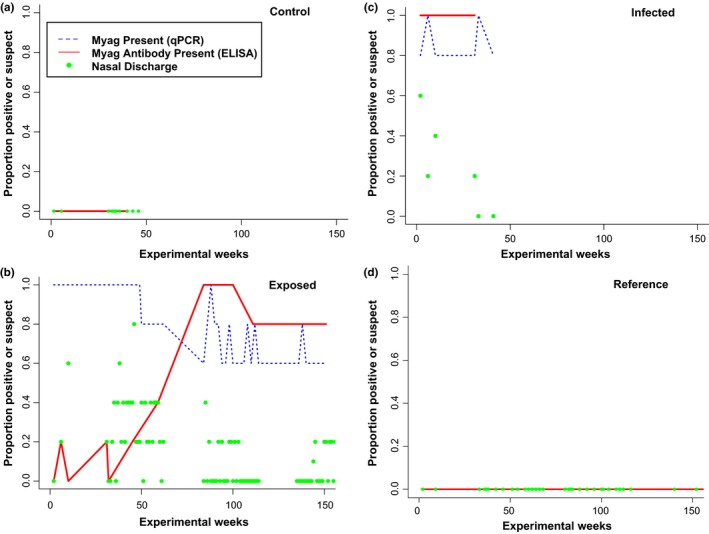
Proportion of Mojave desert tortoises (*Gopherus agassizii*) in each treatment group (control, exposed, infected, reference) with positive or suspect laboratory results for the presence of the pathogen *Mycoplasma agassizii* (Myag via qPCR tests using nasal and oral swabs), Myag antibodies (via ELISA using plasma), and nasal discharge (classified as moderate to severe). *Control *and *reference* tortoises yielded negative laboratory results and did not exhibit nasal discharge. Myag was detected in *exposed* tortoises within 2–4 weeks of exposure, but these animals did not develop Myag antibodies until ~60–85 weeks post exposure. *Infected* tortoises mostly yielded positive results for the presence of and antibodies to Myag throughout the study. Tortoises include five control, five exposed, and five infected captive adult tortoises and 14 reference wild adult tortoises in Clark County, Nevada, USA

Most tortoises in this study had body condition scores within the optimal ranges (range 4–6) indicating adequate muscle and fat deposits relative to skeletal features, with the exception of one infected tortoise “I2” which was classified as underconditioned (BCS 3). Control and reference tortoises were determined to be clinically normal (for physical attributes) throughout most of the experiment and only exhibited recessed eyes or mild clinical findings (e.g. periocular edema). Each tortoise classified as either infected or exposed had multiple significant physical anomalies. Anomalies included periocular edema, conjunctival edema and hyperemia due to inflammation, recession of periocular tissue, ocular and nasal discharge (both serous and mucoid), occluded and eroded nares, labored respiration, pale and reddened oral mucosa and tongue, skin lesions, and associated lethargy. General signs of URTD (e.g. nasal discharge, occluded and eroded nares, periocular edema) were observed in all infected tortoises. URTD signs occurred to a lesser extent among exposed tortoises with increasing time since infection, and were intermittent meaning that the signs presented 1 week and not the following week (Figure 1).

### 3.2 Antibody production

ELISA test results for antibodies specific to Myag were negative for control and reference tortoises and positive for infected individuals throughout the experiment (Figure 1). Exposed tortoises yielded positive ELISA results to Myag after approximately 420–595 days (60–85 weeks) of exposure to infected diseased animals (Figure 1). Most exposed individuals maintained positive antibody serology from summer/fall 2014 until the end of our study (fall 2016). ELISA tests for Myte were negative for control, exposed, and reference tortoises and mostly suspect or positive for infected individuals.

### 3.3 Pathogen detection

PCR test results for nasal swabs, oral swabs, and nasal flushes were negative for all control and freeliving reference tortoises for both Myag and Myte. PCR results for infected and exposed tortoises were mostly positive for Myag, but negative for Myte after exposure to infected animals. For “exposed” tortoises, we detected Myag outside the nasal cavity and inside the mouth and nasal cavity within 30–60 days of introduction to infected animals with consistent positive results in subsequent samples; confirming transmission of this pathogen.

### 3.4 Gene transcription

Due to lack of sufficient RNA, we removed tortoise “I3” from the infected treatment in all transcription analyses. Also, tortoises “E5” and “R12” were removed from the exposed and reference treatments respectively when we evaluated the changes (Δ*C_T_*) in transcription between sampling events, as preexperiment samples were not available. We found no evidence of differences in gene transcript profiles between male and female tortoises in our reference population (*F*_1,61_ = 1.13, *p* = 0.32); therefore, we combined the sexes for analyses.

Given the relatively small sample size within most treatments (~5) and large variation in preexperiment transcript profiles among our treatments (Table 1), we evaluated the changes (Δ*C_T_*) in transcription from our preexperiment sample during each sampling event (Table 2). We found that the changes in transcription (Δ*C_T_*) indicative of immune and physiological function were not statistically different between our experimental treatments (control, diseased, exposed, reference) throughout this study (*sample 2* (*F*_2,12_ = 1.01, *p* = 0.40); *sample 3* (*F*_3,24_ = 1.44, *p* = 0.26); *sample 4* (*F*_3,23_ = 1.80, *p* = 0.10); *sample 5* (*F*_1,13_ = 0.24, *p* = 0.79); Tables 2 and 3). In addition, we found no changes in Δ*C_T_* profiles when we explored the exclusion of reference, infected, and control tortoises for most analyses (Table 3). Only when we evaluated if sick tortoises (infected) differ from healthy wild tortoises (reference), did we find significant differences in Δ*C_T_* during the 4th sampling event when tortoises emerged from brumation in spring (Table 3). This change was largely driven by decreased transcription for genes *ATF*, *CaM*, *CL*, *HSP70*, and *SOD* in infected tortoises (Tables 2 and 3).

**Table 2:** Data range and geometric mean for the change in normalized cycle threshold (Δ*C_T_*) transcription values for 11 genes of interest for 14 adult captive male tortoises and 13 adult wild tortoises (7M:6F) in Clark County, Nevada, USA

Change in cycle threshold (Δ*C_T_*) values									
Gene	Treatment	Postexperiment							
		Sample 2		Sample 3		Sample 4		Sample 5	
		Range	Geo. mean	Range	Geo. mean	Range	Geo. Mean	Range	Geo. mean
AHR	Control	−11.28 to 6.22	−1.39	−2.18 to 4.79	0.92	−3.64 to 2.44	−0.61	—	—
	Exposed	−0.15 to 2.75	0.92	0.01 to 0.58	0.39	0.12 to 1.63	1.00	−2.29 to 2.17	−0.01
	Infected	−1.03 to 0.09	−0.37	−3.13 to 2.08	−0.27	−0.44 to 1.73	0.40	—	—
	Reference	—	—	−2.56 to 2.48	−0.06	−1.84 to 4.21	−0.17	−2.12 to 2.46	−0.20
ATF	Control	−7.74 to 1.47	−1.29	−3.71 to 0.58	1.57	−4.19 to 8.01	−0.08	—	—
	Exposed	−0.80 to 4.27	1.11	−2.13 to 0.95	−0.30	−0.75 to 2.07	0.91	−1.23 to 0.14	−0.49
	Infected	0.03 to 1.05	0.48	−3.42 to 2.84	0.30	1.08 to 4.26	2.26	—	—
	Reference	—	—	−4.48 to 3.82	−1.51	−7.35 to 2.59	−3.30	−2.38 to 6.83	0.83
CaM	Control	−5.6 to 1.15	−0.91	−2.89 to 2.00	−0.83	−2.10 to 3.66	−0.24	—	—
	Exposed		1.20	0.70 to 1.32	0.98	0.19 to 1.29	0.90	−1.99 to 2.21	0.01
	Infected	−0.18 to 1.48	0.40	−1.27 to 2.92	1.29	0.92 to 3.16	1.96	—	—
	Reference	—	—	−4.57 to 2.67	−0.72	−2.91 to 2.36	−0.35	−2.77 to 2.89	0.02
CD9	Control	−3.96 to 1.46	0.14	−3.94 to 1.48	−0.78	−4.55 to 1.60	−1.74	—	—
	Exposed	0.40 to 3.35	1.22	−1.58 to 1.37	−0.69	0.27 to 2.91	1.08	−1.72 to 1.94	0.01
	Infected	−1.16 to 1.10	−0.16	−3.60 to 2.46	−0.62	−0.17 to 3.69	1.38	—	—
	Reference	—	—	−3.65 to 2.16	−0.60	−3.97 to 2.07	−1.04	−3.82 to 3.23	−0.85
CL	Control	−14.44 to 1.36	−4.30	−5.47 to 0.38	−2.72	−4.52 to 10.73	−0.93	—	—
	Exposed	0.14 to 5.12	1.65	−0.68 to 0.74	−0.14	−0.46 to 1.94	0.89	−2.67 to 1.68	−0.31
	Infected	−0.67 to 1.10	−0.03	−4.37 to 3.22	−0.54	−0.01 to 3.45	1.45	—	—
	Reference	—	—	−7.20 to 5.22	−0.56	−3.50 to 1.82	−1.20	−2.66 to 8.61	0.60
HSP70	Control	−8.47 to 2.41	−1.13	−6.00 to 2.36	0.60	−2.44 to 1.71	0.01	—	—
	Exposed	0.18 to 1.73	0.69	0.46 to 1.00	0.67	0.24 to 1.04	0.65	−0.54 to 1.43	0.67
	Infected	0.01 to 1.09	0.47	−0.30 to 1.55	0.99	0.50 to 1.78	1.15	—	—
	Reference	—	—	−1.54 to 2.66	0.29	−1.56 to 1.52	−0.22	−1.07 to 2.26	−0.01
Lep	Control	0.21 to 18.84	4.46	−2.48 to 1.90	−0.07	−2.14 to 0.60	−0.58	—	—
	Exposed	0.35 to 4.55	1.44	0.78 to 1.37	1.07	0.68 to 2.32	1.35	−2.42 to 2.15	−0.05
	Infected	−1.85 to 0.10	−0.90	−2.81 to 3.72	0.03	−0.43 to 2.10	0.66	—	—
	Reference	—	—	−1.79 to 3.26	0.27	−2.85 to 3.22	−0.25	−1.96 to 3.93	−0.73
MyD88	Control	−12.65 to 1.23	−3.15	−2.90 to 1.84	−0.18	−3.65 to 1.34	−0.96	—	—
	Exposed	0.46 to 1.97	0.87	0.19 to 0.74	0.43	0.69 to 2.06	1.11	−1.03 to 2.08	0.44
	Infected	−1.23 to 0.52	−0.26	−2.42 to 1.85	−0.02	−0.05 to 2.51	0.83	—	—
	Reference	—	—	−2.32 to 2.01	−0.40	−1.74 to 3.16	−0.19	−2.57 to 2.42	−0.57
Mx1	Control	−4.08 to 22.21	3.08	−0.12 to 7.57	2.94	−4.76 to 6.34	0.51	—	—
	Exposed	−0.66 to 4.51	1.14	−1.66 to 3.14	1.38	0.91 to 2.55	1.49	−1.80 to 2.99	0.25
	Infected	−4.33 to 0.33	−2.40	−5.07 to 5.50	−0.89	−3.95 to 3.28	−0.95	—	—
	Reference	—	—	−2.93 to 4.22	1.00	−4.48 to 4.75	0.89	−1.10 to 5.20	0.62
SAA	Control	−7.10 to 9.06	1.17	0.31 to 8.11	3.03	−3.83 to 1.85	−0.37	—	—
	Exposed	−1.15 to 4.35	1.32	−2.45 to 1.75	0.10	0.51 to 1.82	1.01	−1.24 to 2.69	0.18
	Infected	−3.84 to 0.52	−1.76	−5.66 to 3.19	−1.47	−4.94 to 2.93	−1.34	—	—
	Reference	—	—	−2.82 to 3.25	1.06	−3.29 to 4.92	0.06	−2.74 to 5.62	0.05
SOD	Control	−1.32 to 1.58	0.65	−4.46 to 1.61	−1.16	−3.56 to 1.65	−1.52	—	—
	Exposed	0.76 to 3.57	1.66	−0.85 to 0.72	−0.06	1.01 to 1.92	1.34	−0.90 to 2.69	0.33
	Infected	−1.09 to 0.95	0.14	−3.61 to 2.05	−0.42	−0.18 to 3.12	1.13	—	—
	Reference	—	—	−5.10 to 2.38	−1.93	−4.91 to 2.28	−2.25	−6.67 to 1.91	−1.82

Note

Tortoises include five control, four exposed, and four infected captive animals and 13 wild reference tortoises. Values for Δ*C_T_* were calculated by substracting the preexperiment value in Jul 2013 (Sample 1) from the *C_T_* values for each gene 20 days post in Sept 2013 (Sample 2), 77 days post in Nov 2013 (Sample 3), 222 days post in Mar 2014 (Sample 4), and 420 days post experiment in October 2014 (Sample 5). Note the smaller the mean value, the higher level of transcript for 11 genes.

—: Information is not available.

**Table 3:** Statistical results for normalized cycle threshold (*C_T_*) transcription values and changes in normalized *C_T_* transcription values (Δ*C_T_*) for 11 genes of interest during the 4th sampling event (222 days post experiment)

	Statistical approach	Gene	Group CEIR		Group CEI		Group CER		Group CE		Group EIR		Group ER		Group IR	
			*χ* ^2^	*p*	*χ* ^2^	*p*	*χ* ^2^	*p*	*χ* ^2^	*p*	*χ* ^2^	*p*	*χ* ^2^	*p*	*χ* ^2^	*p*
*C_T_* values	Univariate	AHR	8.95	0.03**	9.03	0.01**	7.80	0.02**	6.81	<0.01**	0.39	0.82	0.01	0.91	0.52	0.47
		ATF	10.56	0.01**	1.97	0.37	5.14	0.08	0.53	0.46	11.80	<0.01**	6.40	0.01**	8.48	<0.01**
		CaM	2.55	0.47	2.02	0.36	1.50	0.47	0.88	0.35	1.17	0.56	0.54	0.46	0.83	0.36
		CD9	5.07	0.17	2.35	0.31	2.95	0.23	1.84	0.17	4.58	0.10	2.50	0.11	3.31	0.07
		CL	5.07	0.17	3.08	0.21	2.78	0.25	1.84	0.17	3.76	0.15	1.60	0.21	2.88	0.09
		HSP70	5.73	0.13	4.49	0.11	5.24	0.07	3.15	0.08	1.02	0.60	0.90	0.34	1.47	0.23
		Lep	7.52	0.06	9.00	0.01**	6.20	0.05**	6.82	<0.01**	0.33	0.85	0.28	0.60	0.13	0.72
		MyD88	5.02	0.17	4.19	0.12	4.02	0.13	3.56	0.06	1.59	0.45	<0.01	0.96	1.19	0.28
		Mx1	8.87	0.03**	3.56	0.17	7.16	0.03**	3.15	0.08	3.46	0.18	0.28	0.60	0.06	0.81
		SAA	7.29	0.06	4.04	0.13	6.40	0.04**	3.15	0.08	1.70	0.43	0.40	0.53	1.19	0.28
		SOD	8.32	0.04**	3.24	0.20	5.34	0.07	2.45	0.11	8.09	0.02**	5.38	**0.02** **	4.78	0.03**
	Multi‐variate	All genes	*F*_3,25_ = 2.48, *p* = 0.03**		*F*_2,13_ = 0.16, *p* = 0.16		*F*_2,21_ = 2.74, *p* = 0.04**		*F*_1,9_ = 2.10, *p* = 0.14		*F*_2,20_ = 2.53, *p* = 0.04**		*F*_1,16_ = 1.97, *p* = 0.14		*F*_1,16_ = 3.13, *p* = 0.03**	
Changes in *C_T_* values (Δ*C_T_*)	Univariate	AHR	3.76	0.29	1.72	0.42	2.89	0.24	1.50	0.22	3.93	0.14	2.88	0.09	1.70	0.19
		ATF	10.85	0.01**	3.33	0.19	6.83	0.03**	0.96	0.33	9.81	<0.01**	5.52	0.02**	6.15	0.01**
		CaM	5.92	0.12	3.61	0.16	2.34	0.31	2.16	0.14	5.38	0.07	1.70	0.19	4.36	0.04**
		CD9	5.62	0.13	4.33	0.11	3.00	0.22	2.94	0.09	3.80	0.15	1.70	0.19	2.89	0.09
		CL	6.51	0.08	3.12	0.21	3.90	0.14	2.16	0.14	5.77	0.06	3.34	0.07	3.82	0.05**
		HSP70	6.64	0.08	2.20	0.33	2.44	0.30	0.00	1.00	7.57	0.03**	2.88	0.09	5.52	0.02**
		Lep	7.75	0.05**	7.10	0.03**	6.37	0.04**	6.00	0.01**	5.40	0.07	4.36	0.04**	1.38	0.24
		MyD88	5.49	0.14	2.40	0.30	3.72	0.16	1.50	0.22	5.74	0.06	3.84	0.05**	2.88	0.09
		Mx1	1.57	0.67	0.91	0.63	<0.01	1.00	0.00	1.00	1.96	0.38	0.00	1.00	1.70	0.19
		SAA	1.21	0.75	0.91	0.63	0.44	0.80	0.00	1.00	1.61	0.45	0.83	0.36	0.43	0.51
		SOD	10.29	0.01**	4.73	0.09	6.02	0.05**	2.94	0.09	9.52	<0.01**	5.52	0.02**	6.15	0.01**
	Multi‐variate	All genes	*F*_3,23_ = 1.80, *p* = 0.10		*F*_2,12_ = 0.90, *p* = 0.61		*F*_2,19_ = 1.34, *p* = 0.26		*F*_1,8_ = 1.06, *p* = 0.44		*F*_2,18_ = 1.96, *p* = 0.09		*F*_1,14_ = 3.42, *p* = 0.05**		*F*_1,14_ = 3.31, *p* = 0.02**	

Note

Differences among treatment groups including Control (C), Exposed (E), Infected (I), and Reference (R) tortoises were evaluated using a nonparametric rank sums tests (Kruskal‐Wallis) using *χ* ^2^ approximation to assess differences in the means. Transcription profiles were also evaluated using a multivariate approach via permutational multivariate analysis of variation (perMANOVA). Tortoises include five control, five exposed, and five infected captive animals and 14 wild reference tortoises.

** Significance at *α ≤ *0.05.

With limited information on transcription responses in ectotherms, and possible influences of circadian patterns, season, temperature, and other environmental factors (e.g., food and water availability), we felt it was important to also explore the *C_T_* profiles during each sampling event. Mojave desert tortoises in particular have prominent seasonal changes in their metabolism and activity levels. We felt that only evaluating changes in transcription (i.e., Δ*C_T_*) from preexperiment samples collected in midsummer (sample 1), a season of lower metabolic activity for tortoises’ may be difficult to interpret across seasons and years. We found no difference in the *C_T_* profiles between our experimental treatments (control, diseased, exposed, reference) before (*sample 1* (*F*_3,24_ = 1.26, *p* = 0.29) or during the first three months of the experiment (*sample 2* (*F*_2,13_ = 0.70, *p* = 0.86); *sample 3* (*F*_3,25_ = 0.89, *p* = 0.48); Table 1). However, once animals emerged from winter dormancy the following spring (222 days post experiment), we found differences in the transcript profiles among the treatment groups (*sample 4* (*F*_3,25_ = 2.48, *p* = 0.03; Table 1)) with changes in genes *AHR, ATF,* *Mx1*, and *SOD* driving most of this dissimilarity (Table 3). Transcript levels for these genes in early spring were decreased in exposed and infected tortoises and not animals classified as control or reference (disease‐free). The *C_T_* values for these genes in control tortoises following winter dormancy represented a fourfold or more increase in transcription compared to tortoises classified as exposed or infected. During our final sampling event, only exposed and reference tortoises were available for evaluation. We found no difference in C_T_ profiles between these two groups the following fall (*sample 5*; 420 days post experiment; *F*_1,15_ = 1.75, *p* = 0.18).

**Figure 2:**
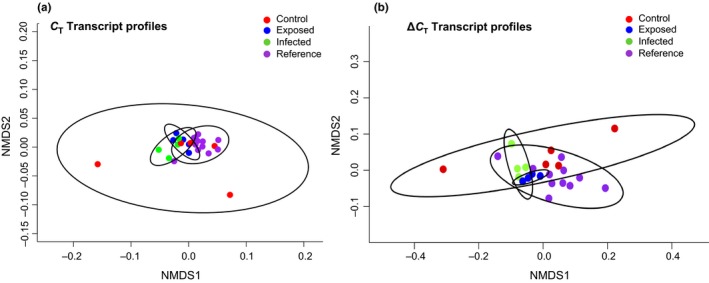
Multivariate, nonmetric multi‐dimensional scaling (NMDS) two‐dimensional plots for (a) normalized cycle threshold (*C_T_*) values for 11 genes of interest in March 2014 (222 days post experiment; a) and the change in *C_T_* values throughout the experiment (222 days–0 days; (b). Blood samples were analyzed for 15 adult captive male Mojave desert tortoises (*Gopherus agassizii*) at the Desert Tortoise Conservation Center and 12 adult (6M:6F) wild tortoises at Hidden Valley in Clark County, Nevada. Tortoises include five control (red), five exposed (blue), five infected (green), and 12 reference (purple) individuals

We also evaluated the *C_T_* profiles when reference and infected tortoises were removed from the analyses. We found no differences in *C_T_* transcription among the groups during early spring (sample 4) when reference and/or infected individuals were excluded from analyses (Table 3). However, we did find significant differences in C_T_ profiles among the treatment groups when control tortoises were excluded, driven mostly by changes in genes *ATF* and *SOD* (Table 3). NMDS and cluster analysis identified significant overall groupings of individuals that were mostly representative of their treatment group in Spring 2014 (sample 4; *C_T_* values); however, less differentiation occurred when Δ*C_T_* profiles were evaluated (sample 4; Figure 2).

## 4 DISCUSSION

While considerable efforts have been directed at understanding the role of infectious diseases such as upper respiratory tract disease (URTD) in the conservation of North American turtles and tortoises (Berry et al., 2015; Brown et al., 1999, 1994; Jacobson et al., 2014; Palmer et al., 2016; Sandmeier et al., 2013), relatively little is known about tortoise immune responses to the causative bacterial agents (Myag and Myte) of this disease (Sandmeier et al., 2012; Sandmeier, Weitzman et al., 2017; Weitzman, Sandmeier, & Tracy, 2017; Zimmerman, Paitz, et al., 2010). We found that infected tortoises were able to transmit Myag to naïve tortoises within 30–60 days by cohabiting within enclosures in seminatural conditions. Clinical signs associated with URTD (e.g., nasal discharge) were routinely present in infected individuals but were slow to develop and variable in exposed tortoises: ranging from rarely to consistently observed depending on the individual. Whereas Myag was detectable inside the oral and nasal cavity of exposed tortoises within 30–60 days, we did not detect an induced antibody response specific to Myag until 420–595 days post exposure, with limited increase in antibody titer for the remaining study. Surprisingly, we found no overall changes in the transcription profiles (Δ*C_T_*) indicative of immune and cellular function among treatment groups (control, exposed, infected, reference) throughout our experiment. Collectively, these findings highlight the complexities in assessing immune function and diagnosing mycoplasmal‐related infections in tortoises.

Ecoimmune studies on endothermic birds and mammals have provided insights on the types and magnitude of responses wild animals have to bacterial pathogens (Hudson, Rizzoli, Heesterbeek, & Dobson, 2002; Martin et al., 2008; Matson, Ricklefs, & Klasing, 2005; Norris & Evans, 2000). Yet, comprehensive studies and laboratory diagnostics are needed to evaluate immune reactions in reptiles and other ectotherms (Flajnik, 1996; Sandmeier et al., 2012; Zimmerman et al., 2009; Zimmerman, Paitz, et al., 2010). Innate immunity for most vertebrates is comprised of a variety of nonspecific molecules and cells that serve as the first line of defense against pathogens (Janeway et al., 2005; Zimmerman, Vogel, et al., 2010). Measures of innate cells and molecules such as lysozymes (Thammasirirak et al., 2006), leukocytes (Christopher, Berry, Henen, & Nagy, 2003; Sandmeier et al., 2016), antimicrobial proteins (Chattopadhyay et al., 2006), natural antibodies (Hunter et al., 2008; Sandmeier et al., 2012), and phagocytic B cells (Zimmerman et al., 2009) have been used to evaluate innate reactions in Mojave desert tortoises and other turtles. However, these studies found the results to be highly context dependent, as patterns of innate immunity were reported to change in response to temperature (Zapata et al., 1992), season (Origgi, 2007; Sandmeier et al., 2016; Zimmerman, Paitz, et al., 2010), and other physiological and environmental factors (Zimmerman, Vogel, et al., 2010).

In contrast to innate immune responses, there is far less information on induced immune reactions such as humoral (antibody) or cell‐mediated (T cell) responses against pathogens in tortoises and other reptiles. Antibody titers to bacterial pathogens are produced in tortoises; however, they typically increase little, do not increase in binding affinity (Zimmerman, Vogel, et al., 2010), and are likely not the primary line of defense. In addition, induced antibodies do not appear to provide protective immunity (Jacobson et al., 2014). Tortoises are routinely screened for mycoplasmal infections using ELISA to measure induced humoral responses to *Mycoplasma* spp. (Schumacher, Brown, Jacobson, Collins, & Klein, 1993; Wendland et al., 2007). We found that infected tortoises consistently produced antibodies to Myag for the duration of study, yet induced antibodies were delayed for exposed tortoises even after Myag was confirmed in the nasal and oral cavity. Once confirmed, Myag‐induced antibody titers in most exposed tortoises remained present for the duration of our study. Extensive surveys of ELISA seroprevalence in captive and wild tortoise populations indicate that Myag‐induced antibodies are frequent and highly variable, generally increase in areas with higher human densities (Berry et al., 2015), occur in most geographic populations in the Mojave Desert, and may be influenced by many life‐history and environmental factors (see review by Jacobson et al., 2014). Our results, and those from prior studies on tortoises, suggest that production of induced antibodies is variable (Origgi, 2007; Zimmerman, Vogel, et al., 2010), influenced by season and other environmental parameters (Martin et al., 2008; Zapata et al., 1992) and can be highly delayed (>18 months) following exposure to pathogenic microbes (Aiello et al., 2016; Maloney, 2011; Sandmeier et al., 2012; Sandmeier, Weitzman et al., 2017).

We questioned why induced antibodies specific for Myte were detected in our infected tortoises, yet we found no evidence of Myte bacteria in these animals. It is possible that the infected tortoises were exposed to Myte and neutralized this infection prior to our experiment. That said, the clearance rates for *Mycoplasma* spp. are unknown in tortoises and are likely negligible (Sandmeier, Weitzman et al., 2017). It is also possible that the pathogen load was very small and swabbing of the nasal and oral cavity did not yield detectable levels of Myte via qPCR. *Mycoplasma* bacteria are known for hiding in the epithelial tissue within its host, particularly in the nasal cavity of tortoises (Jacobson et al., 2014).

### 4.1 Transcript profiling of antibacteria immune responses

We expected molecular reactions to genes responding to pathogenic bacteria and other microbes (SAA, ATF, CD9, MX1, MyD88; Zhou et al., 2008; Zhou et al., 2011; Kibenge et al., 2005; Tumpey et al., 2007; Li et al., 2011) to increase in infected tortoises and individuals exposed to *Mycoplasma* spp. We based this assumption on results of other transcript studies with Chinese soft‐turtles (*Trionyx sinensis*) experimentally infected with the bacterium, *Aeromonas hydrophila *(Zhou et al., 2008, 2011), where turtles exhibited immediate and strong increases (>1,200‐fold) in mRNA transcripts for targeted genes (e.g. SAA, ATF) within two days of exposure, although transcript responses varied in magnitude and duration among the evaluated tissues (Zhou et al., 2008, 2011). In our study, overall changes in transcript profiles for tortoises that were exposed to and infected with pathogenic bacteria were not detected (Δ*C_T_*).

**Figure 3:**
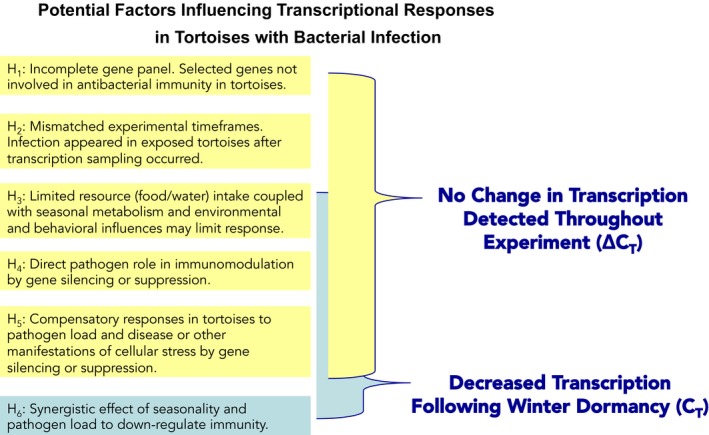
Hypotheses explored to explain why changes in transcription (Δ*C_T_*) for genes thought to be involved in antibacterial immunity were not observed in tortoises exposed to and infected with *Mycoplasma agassizii* throughout our experiment. Evaluation of transcription levels (*C_T_*) for genes following winter dormancy provided an additional hypothesis of a potential synergic effect of seasonality and pathogen load to down‐regulate immunity

Interpreting molecular and physiological data in wild or semi‐wild ectotherms, such as tortoises, often proves difficult. We postulated several reasons why changes in transcription were not observed in tortoises exposed to and infected with *Mycoplasma* bacteria (Figure 3).

**H_1_**: First, we acknowledge that the selected genes in our panel may not be involved with antibacterial immunity in tortoises. Our transcript panel represents a small list of 11 genes with suspected involvement in immune and cellular function (Bowen et al., 2015; Supporting Information Table 1). Recently unveiled genome assemblies for *G. agassizii* revealed 20,172 proteincoding genes (Tollis et al., 2017), and likely hundreds if not thousands of these genes are involved in innate and induced immune responses to pathogenic bacteria with essential, nonessential, or redundant roles (Teglund et al., 1998). We assumed that the function and responses of our selected genes would be fairly conserved across taxa (e.g., Kibenge et al., 2005; Qu, Xiang, & Yu, 2014); however, comparable literature for reptiles and individuals housed in natural environments is largely unavailable.

**H_2_**: We hypothesized that the timeframes associated with our experiment may be mismatched, and that significant infection may not have happened in exposed tortoises until after transcription sampling had occurred. Myag specific antibodies were not detected in exposed tortoises until more than a year after our last complete transcription sampling with all available treatment groups.

**H_3_**: We also speculated that limited food and water intake coupled with seasonal changes in metabolism and environmental and behavior influences would limit transcription responses. The immunosuppressive effects of nutritional deficiencies in vertebrates are well documented (Drake et al., 2016; Iyer, Brown, Whitehead, Prins, & Fairlie, 2015; Saucillo, Gerriets, Sheng, Rathmell, & MacIver, 2014; Weston & Memom, 2009), and although we supplemented captive tortoises with food and water during periods of activity, we did not specifically quantify intake. Previous studies on diseased tortoises found that nasal discharge reduced the sense of smell and hence the ability to locate food (Germano, Zerr, Esque, Nussear, & Lamberski, 2014). It is also plausible that levels of transcription were also modulated by environmental or behavioral factors that were not addressed in this study (Boei et al., 2017). We did not quantify the activity levels, internal body temperatures, or metabolic rates for individual tortoises, but realize that temperature changes can have profound effects on metabolism and immunity by modulating important triggers and regulators of immune pathways (Ferguson, Kortet, & Sinclair, 2018; Sandmeier, Weitzman, & Tracy, 2018).

### 4.2 Pathogen and host dynamics in immunomodulation

Disease pathogenesis in many species is influenced by a multitude of factors providing a complex picture of subtle host‐parasite interactions that result in either control of infection or development of disease. Many mycoplasmal species that infect animals or humans are thought to successfully evade or modulate the host immune response by either intracellular localization or immunomodulatory activity (Burki, Frey, & Pilo, 2015; Finlay & McFadden, 2006). For example, *Mycoplasma *bacteria can create variations in surface antigens using recombinational DNA events of genes (Waites & Talkington, 2004) making it difficult for the host to eliminate its targeted invader. Successful pathogens often use multiple specialized strategies such as programmed cell death, hormone signaling, expression of defense genes, or other basal defenses (Curtin & Sperandio, 2011) to suppress host defense responses and induce disease susceptibility in otherwise resistant hosts.

**H_4_:** In our experiment, we postulated that *Mycoplasma* plays a direct role in silencing or suppressing molecular reactions in infected tortoises. **H_5_**: In addition, it is possible that limited molecular responses in diseased tortoises may also be due to compensatory host responses to pathogen load and disease, or some other manifestation of cellular stress and general overall gene silencing or suppression (Danner, Pai, Wankeri, & Meister, 2016).

A domino effect of molecular signaling and pathway interruption may impede reactions from reaching their desired target. For example, AHR is well known to respond to a diversity of ligands with the induction of expression of many genes and production of different biological and toxic effects (Bonati, Corrada, Tagliabue, & Motta, 2017; Schmidt & Bradfield, 1996). AHR also plays key regulatory roles in a variety of endogenous developmental and immune response processes (Esser & Rannug, 2015) that can affect the binding and activation of targeted proteins such as heat shock proteins. If cytokines and other immune biochemicals interrupt AHR pathways, the activation of associated transcription processes and targeted proteins would likely be affected.

### 4.3 Synergic effects of seasonality and pathogen load to modulate immunity

Evaluating transcriptional changes within individuals over time (Δ*C_T_*) is largely considered the most appropriate approach to measure molecular responses. However, our preexperiment samples were collected in midsummer, a season generally associated with limited activity and reduced metabolic activity for tortoises (Peterson, 1996a, 1996b), and we don't know how changes in metabolism and resource intake during this time influence the ability of genes to be transcribed and expressed. Therefore, we also evaluated the transcription levels (*C_T_*) for each gene during each sampling event. Similarly, we found no changes in transcription before or during the first three months of our experiment. However, once animals emerged from winter dormancy the following spring (222 days post experiment), we found that exposed and infected (diseased) tortoises had decreased transcription for four genes responding to defenses against microbial pathogens (Mx1, Kibenge et al., 2005; ATF; Zhou et al., 2008), cellular and oxidative stress (SOD; Walsh et al., 2010; Sarma & Sharma, 2016), and cell differentiation, growth, and renewal as well as ligand proliferation and activation of protein chaperones (AHR; Stanford et al., 2016; Bonati et al., 2017). General patterns of downregulated transcript profiles have been previously observed in adult tortoises characterized as diseased and ill (Drake et al., 2017) and juvenile tortoises malnourished from invasive plant diets (Drake et al., 2016). However, tortoises in those studies displayed advanced stages of disease or had multiple physiological complications, likely impacting their ability to mount an overall response. These findings do not detract from our early hypotheses, only suggesting that there may also be a synergic effect of seasonality and pathogen load to down‐regulate immunity in tortoises (**H_6_**; Figure 3).

### 4.4 Molecular roles in immunity

Molecular profiling and modeling have made important contributions to understanding how genes involved in immunity are influenced. For example, RNA interference (RNAi), morpholinos, chemical inhibitors and hypomorphic mutations often lead to partial suppression of gene function, whereas null mutations can ablate gene function (Housden et al., 2017). We also know that a host of other factors such as age (Zhang, Drake, Morrison, Oberley, & Kregel, 2002), metabolism (Boei et al., 2017), prior exposure (Louis, Bhagooli, Kenkel, Baker, & Dyall, 2017), and environmental condition (Mangino, Roederer, Beddall, Nestle, & Spector, 2017) can influence these processes. Although pinpointing the specific mechanisms that may control transcription activity in our diseased tortoises is beyond the scope of this paper, our findings provide an opportunity to further explore this phenomenon and highlight the counterintuitive physiological responses often observed in tortoises and other reptiles during disturbance events (authors unpublished work; Sandmeier et al., 2016; Theodorakis et al., 2017).

## 5 CONCLUSIONS

Our research highlights the nuances and complexities associated with diagnosing microbial infections in tortoises, thus contributing to the growing body of literature on the general immune function of ectothermic vertebrates. Comprehensive and integrative assays, and well‐designed experiments are still needed on tortoises in natural environments to accurately evaluate innate immune reactions to exposure to pathogens and disease. Our findings suggest that no single metric, e.g. clinical evaluations, induced ELISA antibody tests, etc. should be used independently to evaluate mycoplasmal infections and disease status in tortoises at this time, especially when only limited survey is possible (Brown et al., 2002; Jacobson et al., 2014; Sandmeier et al., 2009). Improvements in multi‐faceted diagnostic capabilities and longterm surveillance of immunity and overall health will continue to be instrumental in measuring disease impacts to declining reptile populations.

## CONFLICT OF INTEREST

None declared.

## AUTHOR CONTRIBUTIONS

All authors contributed to the design of this study. K.K.D. secured funding, obtained permits, collected field data, performed statistical analyses and wrote the manuscript. C.M.A. secured funding, obtained permits, provided logistical support and collected field data for the captive tortoise experiment. L.B. and S.C.W. conducted the gene transcription laboratory work, provided advice on project design and data analyses, and edited the manuscript. R.L.L. provided conceptual advice on analyses, strategic approaches to the project, and edited the manuscript. T.C.E., K.E.N, and P.J.H. secured funding, obtained permits, provided conceptual advice on project design and analyses and edited the manuscript.

## Data Availability

Gene PCR primers for our gene transcription panel can be found in Bowen et al. (2015). Ecological and disease data for this experiment can be found at http://doi.org/10.5066/P940J8EY.
