# Supplementary material for: Complex immune responses and molecular reactions to pathogens and disease in a desert reptile (Gopherus agassizii)
Source: Ecol Evol. 2019 Feb 18;9(5):2516–34. doi: 10.1002/ece3.4897 (PMC6405529; doi:10.1002/ece3.4897)
Supplement: Supplementary file 1 [file ECE3-9-2516-s001.docx]

**Supplemental Table**

| **Gene** | - - - 1. **Gene function** |
| --- | --- |
| CaM | Calmodulin (CaM) is a small acidic Ca^2+^-binding protein, with a structure and function that is highly conserved in all eukaryotes. CaM activates various Ca^2+^-dependent enzyme reactions, thereby modulating a wide range of cellular events, including metabolism control, muscle contraction, exocytosis of hormones and neurotransmitters, and cell division and differentiation (Chen et al. 2012). CaM has also been reported to be a pivotal calcium metabolism regulator in the shell formation (Li et al. 2004). |
| AHR | The arylhydrocarbon receptor (AHR) responds to classes of environmental toxicants including polycyclic aromatic hydrocarbons, polyhalogenated hydrocarbons, dibenzofurans, and dioxin (Oesch-Bartlomowicz et al. 2005). Depending upon the ligand, AHR signaling can modulate T-regulatory (T_REG_) (immune-suppressive) or T-helper type 17 (T_H_17) (pro-inflammatory) immunologic activity (Quintana et al. 2008, Veldhoen et al. 2008). |
| Mx1 | The Mx1 gene responds to viral infection (Tumpey et al. 2007). Vertebrates have an early strong innate immune response against viral infection, characterized by the induction and secretion of cytokines that mediate an antiviral state, leading to the up-regulation of the MX-1 gene (Kibenge et al. 2005). |
| HSP 70 | The heat shock protein 70 is produced in response to thermal or other stress (Iwama et al. 1999, Tsan and Gao 2004). In addition to being expressed in response to a wide array of stressors (including hyperthermia, oxygen radicals, heavy metals, and ethanol) heat shock proteins act as molecular chaperones (De Maio et al. 1999). For example, heat shock proteins aid the transport of the AHR/toxin complex in the initiation of detoxification (Tanabe at al. 1994). |
| SAA | Serum Amyloid A (SAA), an acute phase protein, serves as a core part of the innate immunity involving physical and molecular barriers and responses (Cray et al. 2009). Upon infection and inflammation or tissue damage and stress, SAA is induced by pro-inflammatory signals, and is a major indicator of bacterial infection, especially at early stage, in reptiles (Zhou et al. 2008, 2011). |
| MyD88 | Myeloid differentiation factor 88 (MyD88) is one of the key adaptor proteins to signal transduction that triggers downstream cascades involved in innate immunity. MyD88 might possess an important role in defense against microbial infection in Chinese soft-shelled turtles similar to that in mammals (Li et al. 2011). |
| CD9 | CD9 is a molecular facilitator, provides co-stimulation to naïve T lymphocytes, regulates the aggregation of MHC-II molecules, and triggers antigen presentation. Upregulation of turtle CD9 was shown in response to bacterial infection (Zhou et al. 2008, 2010). |
| SOD | Superoxide dismutase (SOD) – Superoxide dismutases are a class of enzymes that catalyze dismutation of superoxide into oxygen and hydrogen peroxide and function as important antioxidant defense molecules (Walsh et al. 2010). Oxidative stress itself can lead to or result from certain inflammatory conditions (Walsh et al. 2010). |
| ATF | Similar to inflammatory responses in mammals, ATF can mediate inflammatory responses in reptiles. The upregulation of ATF in reptiles indicates involvement in bacterial infection (Zhou et al. 2008). |
| CL | Cathepsin L, an acute phase protein, plays a major role in antigen processing, tumor invasion and metastasis, bone resorption, and turnover of intracellular and secreted proteins involved in growth regulation (Zhou et al. 2008). Turtle cathepsin L may be involved in anti-bacterial immune response (Zhou et al. 2008). |
| LEP | Leptin links nutritional status with neuroendocrine and immune functions. Initially thought to be a satiety factor that regulates body weight by inhibiting food intake and stimulating energy expenditure, leptin is a hormone whose multiple effects include regulation of endocrine function, reproduction, and immunity (Otero et al. 2005). |

Supplemental Table 1. Recognized functions of 11 genes identified in adult Mojave desert tortoises (*Gopherus agassizii*).
